# Effect of perioperative cognitive behavioural therapy on chronic post-surgical pain among breast cancer patients with high pain catastrophising characteristics: protocol for a double-blinded randomised controlled trial

**DOI:** 10.1186/s13063-022-06019-z

**Published:** 2022-01-21

**Authors:** Aneurin Moorthy, Damien Lowry, Carla Edgley, Maire-Brid Casey, Donal Buggy

**Affiliations:** 1 Division of Anaesthesiology & Perioperative Medicine, Mater University Hospital, Dublin, Ireland; 2 Depts of Psychology and Pain Medicine, Mater Misericordiae University Hospital, Dublin, Ireland; 3 School of Medicine, University College Dublin, Dublin, Ireland; 4 Mater Misericordiae University Hospital, Dublin, Ireland; 5 Division of Anaesthesiology & Perioperative Medicine, Mater University Hospital, School of Medicine, University College Dublin, Dublin, Ireland; 6 Outcomes Research, Cleveland Clinic, Cleveland, OH USA

**Keywords:** Breast cancer surgery, Chronic postsurgical pain, Mastectomy, Cognitive behavioural therapy, Psychological factors, Pain catastrophizing, Anxiety

## Abstract

**Background:**

Surgery is regarded as the primary treatment for breast cancer. Chronic post-surgical pain (CPSP) is a recognised complication after breast cancer surgery, and it is estimated to affect 20–30% of women. Pain catastrophizing has emerged as one of the most influential psychological variables associated with CPSP.

**Methods:**

This trial will be a single-centre, prospective, double-blinded, superiority, randomised controlled trial (RCT). Patients scheduled for elective breast cancer surgery (wide local excision or mastectomy with or without axillary lymph node dissection) will be screened preoperatively for high pain catastrophising. Patients with high pain catastrophising, defined as a score of ≥ 24 on the Pain Catastrophising Scale will be deemed eligible for inclusion in the study. Participants will be randomly assigned to receive either a cognitive behavioural therapy or an educational mindfulness based programme during their perioperative period. The primary outcome is the Brief Pain Inventory short form average pain severity score at 3 months postoperatively. Secondary outcomes include patient-reported quality of recovery at days 1–2 after surgery, levels of pain catastrophising, reported depressed mood and anxiety.

**Discussion:**

To the best of our knowledge, this protocol describes the first RCT which directly examines the effect of perioperative cognitive behavioural therapy on CPSP among breast cancer patients with high pain catastrophising characteristics. The outcomes of this trial may have significant implications for these patients because perioperative cognitive behavioural therapy has the potential to become an important perioperative intervention to complement patient management.

**Trial registration:**

ClinicalTrials.govNCT04924010. Registered on 11 June 2021. All item from the World Health Organisation Trial Registration Data set have been included.

**Supplementary Information:**

The online version contains supplementary material available at 10.1186/s13063-022-06019-z.

## Administrative information

Note: the numbers in curly brackets in this protocol refer to SPIRIT checklist item numbers. The order of the items has been modified to group similar items (see http://www.equator-network.org/reporting-guidelines/spirit-2727-statement-defining-standard-protocol-items-for-clinical-trials/).

**Table:** 

Title {1}	Effect of perioperative cognitive behavioural therapy on chronic postsurgical pain among breast cancer patients with high pain catastrophising characteristics: study protocol for a double-blinded randomised controlled trial.
Trial registration {2a and 2b}.	This trial was pre-registered on ClinicalTrials.gov on 11 June 2021. Identifier number: NCT04924010. All items from the World Health Organisation Trial Registration Data set have been included. https://clinicaltrials.gov/ct2/show/NCT04924010
Protocol version {3}	Protocol version 2: 05 February, 2021
Funding {4}	This trial is being funded internally from the division of Anaesthesiology & Perioperative Medicine, Mater University Hospital. No external funding sourced.
Author details {5a}	(1). Dr Aneurin Moorthy*: Anaesthesia research fellow, Division of Anaesthesiology & Perioperative Medicine, Mater University Hospital, Dublin, Ireland; aneurin.moorthy@gmail.com (Correspondence). *Co-first author (2). Dr Damien Lowry*, Chartered Senior Counselling Psychologist, Depts of Psychology and Pain Medicine, Mater Misericordiae University Hospital. *Co-first author (3). Dr Carla Edgley, University College Dublin, School of Medicine. (4). Dr Maire-Brid Casey, Senior Physiotherapist, Mater Misericordiae University Hospital. (5). Professor Donal J. Buggy**: Consultant Anaesthesiologist, Division of Anaesthesiology & Perioperative Medicine, Mater University Hospital, School of Medicine, University College Dublin, Ireland; and Outcomes Research, Cleveland Clinic, OH, USA. donal.buggy@ucd.ie. **Principal investigator
Name and contact information for the trial sponsor {5b}	Division of Anaesthesiology & Perioperative Medicine, Division of Anaesthesiology & Perioperative Medicine, Mater University Hospital, Dublin, Ireland. Email: anaes@mater.ie, Office: 003531803 2286/2281
Role of sponsor {5c}	This is a hypothesis-driven, investigator-initiated trial. Therefore, the funders played no role in the design of the study, data collection, analysis, interpretation of data or in the writing of the manuscript.

## Introduction

### Background and rationale {6a}

Chronic post-surgical pain (CPSP) is defined as pain at or near the site of surgery persisting for 3 months or more after the date of surgery. The incidence of CPSP in Europe is up to 50% at 3 months and 12% at 12 months, but the incidence varies depending on surgical procedure [1]. In breast surgery, one of the most commonly performed surgical procedures for cancer [2], CPSP after breast cancer surgery has been observed in 28% at 3 months [3] and up to 20–30% of patients at 6 months after surgery, making this group among the highest risk of developing CPSP [4, 5]. Clinical developments that could mitigate the development of CPSP following breast cancer surgery would potentially yield multiple benefits in terms of reducing future healthcare utilisation, associated costs [4, 6, 7], and improving physical and mental health outcomes in this patient cohort [3, 8].

Several predictive factors for CPSP have been identified, the most important being the presence of chronic pre-operative pain, high intensity of acute postoperative pain and several psychological factors [9]. Of these psychological factors, pain catastrophizing has emerged as one of the strongest predictors of pain severity and disability among individuals with a range of pain presentations and CPSP [10–13]. Pain catastrophizing is described as a maladaptive psychological coping strategy involving an exaggerated reaction to anticipated or actual pain. It can involve mental rumination, magnification of the perceived danger or threat associated with pain and feelings of helplessness in relation to what can be done [10, 13]. A recent systematic review on psychological interventions in patients undergoing major elective abdominal surgery concluded that pain catastrophising can have a direct influence on the neuropathophysiological mechanisms underlying pain experiences and can worsen pain and psychological outcomes, after surgery [14].

Psychological variables are modifiable, and catastrophizing appears to be an exciting clinical target for intervention. In recent years, there have been a growing number of studies investigating the potential impact of perioperative psychological interventions in a variety of patient groups. A recent systematic review and meta-analysis of observational studies concluded that psychological predictors may have a significant association with chronic post-surgical pain, including catastrophisation, although this conclusion is limited by the heterogeneity of study designs, methods used and a lack of robust randomised controlled trial (RCT) data to help delineate causative links [15].

### Objectives {7}

The primary objective of this RCT is to examine whether a perioperative cognitive behavioural therapy (CBT)-based psychological intervention is effective at reducing chronic pain intensity at 3-month follow-up, in high catastrophising patients undergoing breast cancer surgery, as compared to a pain education and mindfulness programme.

Secondary objectives of the study include examining whether perioperative CBT has an impact in reducing pain interference, quality-of-recovery, pain catastrophising, depression and anxiety compared to a pain education and mindfulness programme.

### Trial design {8}

This is a single-centre, prospective, double-blinded, superiority, randomised controlled trial (RCT).

**Fig. 1:**
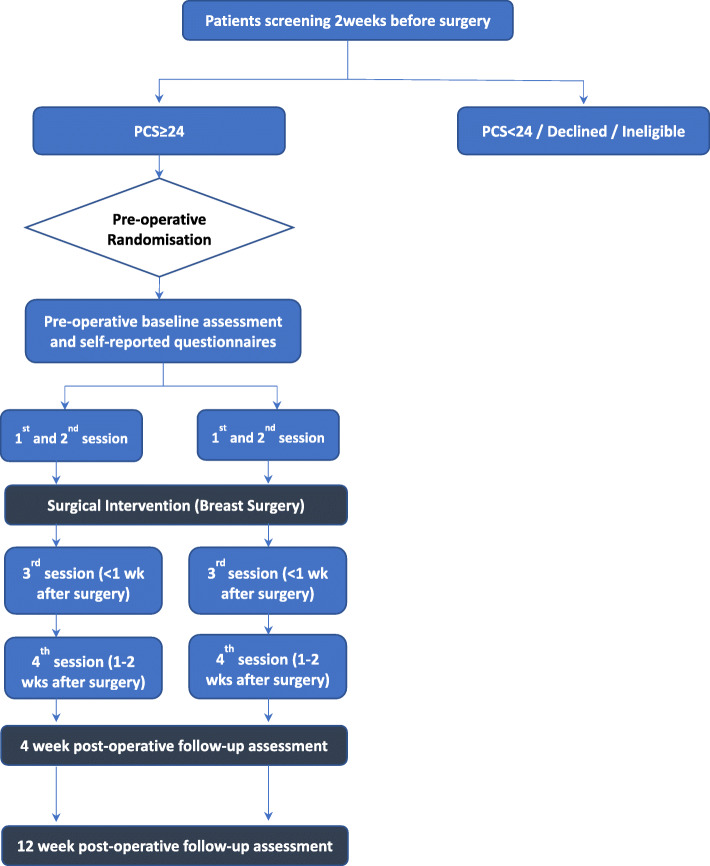
Study flow chart

The study will randomise breast cancer surgery patients into two groups. One group will receive four sessions of perioperative cognitive behavioural therapy (CBT) in addition to usual care, and the other group will receive four perioperative sessions of pain education and mindfulness exercises. Figure 1 illustrates the study flow.

## Methods: participants, interventions, and outcomes

### Study setting {9}

This study will be conducted in a tertiary university hospital in Dublin (Mater Misericordiae University Hospital), Republic of Ireland. The hospital performs over 500 breast cancer surgeries per year.

### Eligibility criteria {10}

Inclusion criteria are:

- Female patients
- Aged 18–75
- Undergoing breast cancer surgery (either wide local excision with magseed or full mastectomy)
- Pain Catastrophising Scale (PCS) score of 24 or higher

Exclusion criteria are:

- Surgery for benign breast disease
- Patient non-consent
- Plans to undergo major surgery within three months after current breast surgery
- Comorbid severe psychiatric conditions such as schizophrenia or personality disorder
- Known or suspected non-compliance
- Known or suspected drug or alcohol abuse problems within past 3 months
- Inability to follow the study procedures e.g. dementia or non-fluency of English
- The presence of any serious medical comorbidity that might cause disability or worsen the patient’s general health condition
- Pregnancy
- An opioid intrathecal pump
- Cognitive behavioural therapy in the past 12 months.
- Inability to complete at least one psychological session prior to breast cancer surgery

### Who will take informed consent? {26a}

The senior pain psychologist (DL) delivering the perioperative interventions for this trial will screen for potential trial participants, after breast cancer surgery clinic nurses identify potential candidates for the study. Patients that meet the eligibility criteria will be contacted, and informed consent will be obtained. All potential participants will have the opportunity to withdraw at any time point during the study period.

### Additional consent provisions for collection and use of participant data and biological specimens {26b}

Not applicable. No data and biological specimens will be collected for use in ancillary studies

## Interventions

### Explanation for the choice of comparators {6b}

CBT is a form of psychological treatment that is widely used to treat various mental health disorders. We aim to examine its effectiveness in reducing pain intensity and development of chronic post-surgical pain (CPSP) 3 months post-breast cancer surgery. To achieve this, we have designed our trial to compare CBT with an active control arm, a pain education and mindfulness programme. By taking this approach, the study is controlled for the following factors: time exposure, attention the patient receives with the treating psychologist and pain education. It will be controlled for pain education because this element will be emphasised to the same degree in both the CBT-CP and PEM groups. This will help to specifically isolate the potential effects of CBT on those participants in that arm of the study.

### Intervention description {11a}

The two study interventions for this trial are Cognitive Behavioural Therapy for Chronic Pain (CBT-CP) and pain education and mindfulness (PEM). Both of these interventions will be delivered by the same senior psychologist, with 17 years of hospital psychology experience including seven in the treatment of chronic pain patients. If the treating psychologist is not available to implement the study interventions, then active recruitment of patients for this trial will temporarily come to a halt. Patient enrolment will resume once the assigned treating psychologist is made available. The purpose of this is to maintain homogeneity and consistency regarding delivery of the study interventions.

## Group 1: Cognitive Behavioural Therapy for Chronic Pain (CBT-CP)

The CBT intervention will be delivered as individual therapy appointments to patients and will last for 60 min per session. During these sessions, there will be an emphasis on mindfulness based stress reduction, cognitive restructuring, exercise and pacing, behavioural activation, improving sleep and anger management. Standardised worksheets and homework assignments are an important part of CBT-CP, and these will be given to patients who will be asked to read and complete in terms of complementing the consultations. A total of four sessions will be delivered during the perioperative period, with at least one of these taking place prior to surgery. Further details of each individual session and its content are provided in Table 1. This intervention has been developed with reference to two sources: a successful national implementation of CBT-CP using video teleconferencing format [16] and an evidence-based CBT manual specifically designed to treat chronic pain [17].

**Table 1:** Summary of key components of cognitive behavioural therapy sessions delivered pre and post-surgery

Timing	Aim(s)	Content
**Pre-surgery**		
Session 1: 1–2 weeks pre-surgery 60 min Video-teleconference	Establish rapport Discussing treatment rationale Pain education Relaxation methods	Introductions Gather personal history info Discuss pain education re: relations between cognition, emotions, behaviour and consequences Introduce concept of fear avoidance Build awareness on the potential impact of stress and pain experience Practice relaxation methods (PMR) **Homework:** Monitoring cognitions, emotions and consequences; identify ‘challenging’ moments; practice PMR
Session 2: 1–3 days pre-surgery 60 min Video-teleconference	Discuss surgery and associated fears/emotions/cognitions Validate these fears and support person’s emotional state Focus discussion on any catastrophizing thoughts (e.g. “what if it goes wrong…,” “something bad might happen…,” etc.	Homework review Relaxation practice Defining ‘catastrophizing’ and ‘state anxiety’ linking to any relevant homework material Explaining their importance in context of surgery and recovery from Practicing the identification of anxiety and catastrophizing thoughts Practicing cognitive restructuring (challenging and replacing catastrophic thoughts with more adaptive/helpful/constructive ‘facts’) End with PMR exercise **Homework:** PMR; continued mental monitoring and cog restructuring
**Post-surgery**		
Session 3: 1–3 days post-surgery 60 min Video-teleconference	Support participant post operatively Validate their reported state of health/any concerns Maintain conversational focus on catastrophizing tendencies, identifying them as they arise and reinforcing the ability to challenge, restructure, etc.	Homework review Discussion of catastrophizing tendency perioperatively and its links to consequences Discuss potential for fear avoidance and link to consequences Discuss goals and barriers Framing conversation around adaptive coping versus maladaptive coping Acknowledge challenges End with PMR exercise **Homework:** continued PMR practice; continued mental monitoring to detect and challenge catastrophizing cognitions
Session 4: 1–2 weeks post-surgery 60 min Video-teleconference	Check-in Maintaining progress Promoting continued practice	Homework review Note and discuss progress Validate scale of the experience Contextualise content of thought processes and encourage continued cognitive restructuring in situations of ‘catastrophisation’ Reinforce continued PMR methods Discuss medium term goals and barriers Reviewing and summarising main learning points from the last few weeks

## Group 2: Pain education and mindfulness (PEM)

To control for the potential effects of additional time spent with a professional, pain education and mindfulness input between the two interventions, patients in the education and mindfulness group will also have four perioperative psychology sessions, and at least one of which will take place before the surgery. As the same person will be providing the CBT-CP and educational interventions, this potential confounding factor will also be controlled for. The control intervention consists of discussing pain education, as derived from the self-management section of the chronic pain Ireland website (https://www.chronicpain.ie/our-services/self-management ) and the persistent pain section of the pain toolkit website (https://www.paintoolkit.org/persistent-pain). In addition, the control intervention will consist of mindfulness-based stress reduction exercises that will be completed during each session, and emphasis on mindful breathing, guided meditation, and progressive muscle relaxation techniques will be made. Participants in this arm will also be signposted to online mindfulness resources including the wellbeing section of the Mater Hospital website (www.mater.ie/wellbeing) and the Mindfulness and Relaxation Centre resource on a neighbouring hospital’s website’s psychology department section (http://www.beaumont.ie/marc). These resources offset the additional worksheets and resources provided to those in the CBT arm of the study. Like the CBT-CP group, each PEM session will last for a total of 60 min.

### Criteria for discontinuing or modifying allocated interventions {11b}

Criteria for discontinuing the allocated interventions are:

1. Patient withdrawal of consent at any point of study
2. Worsening mental health or psychological well-being of patient

### Strategies to improve adherence to interventions {11c}

All participants for this trial will receive a total of four psychological based interventions (see the ‘Intervention description {11a}’ section). Each intervention session lasts for 60 min. To ensure patient adherence to the study interventions during the trial period, all participants will be well-informed during the consent process. The study participation burden, such as duration and timing of each psychology session, and completion of primary outcome questionnaires at 1 and 3 months post-surgery will be explained. The senior pain psychologist responsible for screening potential study participants will record all eligible candidates and those that do not proceed with the study due to ineligibility, non-consent, or withdrawal. In so far as it is possible, only eligible and fully willing individuals will proceed to randomisation and allocation of the patient to either the CBT-CP or PEM group.

### Relevant concomitant care permitted or prohibited during the trial {11d}

All patients will receive standard perioperative surgical and anaesthesia care during this trial. These include:

1. Pre-operative assessment: This may involve optimisation of underlying medical conditions (e.g., blood pressure or glucose control)
2. Intra-operative interventions: All patients will receive a general anaesthesia as part of their surgical management. This may be complemented with regional anaesthesia techniques (e.g., PECS II block) for post-operative pain management; this will be under the discretion of the treating anaesthesiologist
3. Post-operative interventions: All patients will have a post-operative analgesia plan. Again, this will be under the discretion of the treating anaesthesiologists. Standard post-operative analgesia that may be prescribed but are not limited to include paracetamol, non-steroidal anti-inflammatory drug, morphine, fentanyl and gabapentin

### Provisions for post-trial care {30}

All participants that are enrolled into this study are covered by indemnity for negligent harm, through the standard Health Service Executive (HSE) indemnity arrangements. If any participant suffers from any stress or mental health complications arising directly from either intervention during or after the trial, then the participant will be offered further psychological management in line with standard care. This will be offered by a different clinical psychologist that has no direct or indirect involvement with this trial. The research team for this study will liaise with clinical staff attached to the breast cancer surgery teams to make these arrangements.

### Outcomes {12}

Primary outcome:

1. Brief Pain Inventory Short form: average pain severity score. [time frame: 3 months postoperative]
  Brief Pain Inventory (BPI) short form will be used to assess for CPSP after breast cancer surgery. This assessment will be conducted by a member of the research team. The BPI assesses for quality of life and pain, and its scale is measured between 0 and 10, where ‘0’ indicates no pain and ‘10’ indicates severe pain. A decrease in the BPI score of 2 or more from the baseline score is considered clinically significant and indicates an improvement in severity of the patient’s cancer and or non-cancer pain [18]

Secondary outcomes:

Secondary outcomes of the study include pain interference, quality of recovery from surgery, levels of pain catastrophising, reported depressed mood and levels of anxiety. These will be measured as follows:

- Brief Pain Inventory Short form: average pain interference score. [time frame: 3 months postoperative]
  i. BPI average pain interference score assess for interference the pain has on the patient’s functioning. BPI is measured between 0 and 10, where ‘0’ indicates no interference and ‘10’ indicates severe interference with quality of life [18].
- Quality of Recovery-15 (QoR-15) [time frame: 24–48 h, post-operative]
  - QoR-15 is a 15-item questionnaire which is used as a tool to assess overall patient recovery and pain after surgery. Participant will be asked to complete this questionnaire within 24–48 h after their surgery. It is scored between 0 and 150, where the greater the number indicates excellent post-operative recovery [19].
- Pain Catastrophising Scale (PCS). [time frame: 1 and 3 months, post-operative]
  - PCS is a 13-item questionnaire designed to measure levels of pain-catastrophizing. The scale comprises 13 items which yield an overall catastrophising score, which is a composite of magnification, rumination and helplessness subscales. An overall score of greater than 24 is significant for pain catastrophising [10, 13, 20]
- Hospital Anxiety and Depression Scale (HADS). [time frame: 1 and 3 months, post-operative]
  - HADS is a 14-item self-report questionnaire that was developed and found to be a reliable for detecting states of depression and anxiety in hospital patients, including those with cancer. A depression or anxiety score of greater than 10 is considered significant [21]

### Participant timeline {13}

Time schedule of enrolment, interventions, assessments and visits for participants are illustrated in Table 2.

**Table 2:** Time schedule for various points during study period

		Study period								
	Enrolment	Allocation	Post-allocation						Primary and secondary outcomes	
Time point	*-t*_*1*_	0	14 days pre-op	7 days pre- op	Intra-operative	24-48 h post-op	7 days post- op	14 days post- op	30 days	90 days
**Enrolment:**										
**Eligibility screen (inclusion and exclusion criteria)**	X									
**Informed consent**	X									
**Baseline PCS (> 24: eligible to enrol)**	X									
**Randomisation**		X								
**Interventions:**										
**CBT session group**			X	X			X	X		
**PEM session group**			X	X			X	X		
**Assessments:**										
**Patient demographics and characteristics**	X									
**Intraoperative data**					X					
**BPI**		X							X	X
**QoR-15**						X				
**PCS**	x								X	X
**HADS**									X	X

*PCS* Pain Catastrophising Scale, *CBT* cognitive behavioural therapy, *PEM* pain education and mindfulness, *BPI* Brief Pain Inventory score, *QoR-15* Quality of Recovery-15 score, *HADS* Hospital Anxiety and Depression Scale

### Sample size {14}

The primary outcome is the difference in the BPI average pain severity score between the study groups, three months after surgery. A clinically important difference on the BPI is 2 raw score reduction on the 11-point Numerical Pain Rating Scale (NRS) [22–24]. The standard deviation (SD) of BPI scores after breast surgery is in the order of 2.3 [25] on this scale. Taking a BPI score reduction of 2 as the minimal clinically relevant difference, then *n* = 21 patients would be required each arm if type I error = 0.05 and type II error is 0.2 (power 80%). This calculation was verified by using an online power calculator (https://www.sealedenvelope.com/power/continuous-superiority/). The screening process for potential eligible participants will be a very thorough methodological process. As mentioned in the exclusion criteria, any suspected non-compliant participants will not be enrolled or randomised to either of the study’s intervention. By taking this approach, we endeavour to limit the number of dropouts to approximately 10%, and therefore, we propose to enrol 24 eligible patients in each group (*N* = 48 total).

### Recruitment {15}

Patients eligible for participation will be evaluated approximately 2 weeks before surgery and asked for informed consent, and then asked to complete the Pain Catastrophising Scale (PCS) questionnaire [10, 13, 20] by a member of the research team. Patients with high pain catastrophising, defined as a score of ≥ 24 on the PCS, and who satisfy all other eligibility criteria, will proceed for randomisation to either perioperative CBT or PEM groups. This cutoff is based on a previous study that showed a pre-treatment score of 24 or higher on the PCS best predicted follow-up chronic pain ratings and work status after multidisciplinary treatment [26] and has since been selected as an appropriate score cutoff in a perioperative CBT intervention in lumbar surgery patients [27]. More recently, this cutoff was also suggested to obtain the highest sensitivity and specificity to predict unfavourable outcomes after spinal surgery [28].

### Assignment of interventions: allocation

#### Sequence generation {16a}

Patients will be randomised after they are included in the study, having consented, and completed the PCS (and scoring scored ≥ 24), and two preoperative appointments will be scheduled with the patients in the intervention and control groups. Patients will be randomised to either ‘CBT-CP’ or ‘PEM’ groups by using an online computer-generated block randomisation.

#### Concealment mechanism {16b}

Patient study number and group allocation will be typed onto separate pages and concealed in sequentially numbered sealed opaque envelopes.

#### Implementation {16c}

The randomisation process will be performed by an independent third party. The randomisation key/seed will also be held by an independent third party, and investigators will not have access to this key/seed until the study has been completed, except for the treating psychologist (DL) assigned to this trial. DL will allocate patients to either study intervention only after opening the sealed envelopes prospectively as participants are enrolled. DL will not have any additional involvement in data collection or analysis.

### Assignment of interventions: blinding

#### Who will be blinded {17a}

The treating psychologist will not be blinded as he is required to deliver both interventions associated with this trial. All other members of the research team involved in data collection and analysis will be blinded to the group allocation. In addition, patients will also be blinded because they will not be informed if they are receiving CBT or PEM other than that they are receiving one of two types of psychological interventions.

#### Procedure for unblinding if needed {17b}

Unblinding of trial participants to fellow research team members will occur only after creation of a final locked analysis dataset when the last patient has provided data at 3-month follow-up and after data has been statistically analysed by (MBC and DB).

### Data collection and management

#### Plans for assessment and collection of outcomes {18a}

There will be three time points in which data collection will occur (preoperative, intraoperative, and postoperative). Data will be obtained from electronic and paper patient records. In addition, various questionnaires will be used to gather data to assess for the primary and secondary outcomes outlined for this trial (see the ‘Outcomes {12}’ section).

#### Plans to promote participant retention and complete follow-up {18b}

During the screening process, potential participants will receive an extensive patient information leaflet about the study. The senior pain psychologist involved in this study will emphasise the importance of patient participation and the expectation to complete the study interventions and the various follow-up questionnaires at the time of screening and enrolment to optimise retention and meaningful data collection.

### Data management {19}

All data will be recorded on a study specific case report form (CRF) that has the patient’s unique study identifier code on each page. Study investigators will enter the data from CRF’s into a designed study specific REDCap database that is password protected. Data will be entered primarily by one study investigator (AM) and verified by a different study investigator (CE) to minimise data entry errors (e.g., incorrect, or duplicate data). Both researchers will be blinded to group allocation.

The data collected and all the research-related documents (both hard copies and electronic copies) will be stored securely in a locked office in the principal investigator’s office at the Mater Misericordiae University Hospital (MMUH). Only the principal investigator and the co-investigators can have access to these documents. The records will be kept for 5 years following study closure. All electronic files will be encrypted and accessed via password protected computers.

The electronic REDCap database also allows for specified ranges and automatic calculations to reduce entry errors. The study REDCap database will have automatic calculations for study questionnaires and specified ranges entered for each questionnaire response to ensure accurate data entry. Data will be cleaned by investigators upon completion of data collection to ensure good quality data.

### Confidentiality {27}

All the data collected will remain anonymous and confidential. A unique subject number will be provided to each individual patient participating in the study. The front page of the CRF which will be labelled with both the randomisation number and patient information will be stored separately to the remainder of the CRF containing data about the patient to ensure data is re-identifiable. Only study investigators will have access to the data as CRF forms will be stored in a locked office that only study investigators have access to.

### Plans for collection, laboratory evaluation and storage of biological specimens for genetic or molecular analysis in this trial/future use {33}

Not applicable, no samples will be collected.

### Statistical methods

#### Statistical methods for primary and secondary outcomes {20a}

Outcome analyses will be conducted by researchers (MBC and DB) who will be blinded to treatment group allocation. Descriptive statistics will be calculated for all outcome measures at each time point, including for continuous variables: means, standard deviations or medians with ranges of scores, and for categorical variables: frequencies and percentages.

Descriptive and inferential statistics will be obtained using the appropriate statistical methods required to address the study objectives. The primary analysis will compare the effect of the interventions on the primary outcomes; average pain severity at 3 months post-surgery. Outcome analyses will be conducted according to an intention to treat principle, i.e. all randomised participants will be included in the main analysis and will be analysed as randomised, regardless of protocol adherence. Secondary analysis will involve the analysis of the secondary outcomes: QoR-15 at 24 h post-surgery, PCS, HADS and average pain interference on the BPI short form at 3 months post-surgery. Linear mixed models on the outcome measures over time will be fitted to evaluate the effectiveness of both interventions, which intrinsically adjusts for baseline scores. Statistical significance will be assessed from a *p*-value < 0.05 from the group by time interaction term. For all tests, 2-sided *p*-values will be used, which will be reported to 4 decimal places with *p*-values less than 0.001 reported as *p*< 0.001. In the case of a significant result, contrasts of the group effects at each assessment time point will be used to investigate the direction and pattern of effects. Irrespective of statistical significance, the mean changes and confidence intervals will be reported. An up-to-date version of SPSS will be used to conduct analyses.

#### Interim analyses {21b}

No interim analyses will be conducted.

#### Methods for additional analyses (e.g. subgroup analyses) {20b}

A per-protocol analysis will exclude participants found to be ineligible after randomisation and those who did not receive the intervention. Both intention to treat and per-protocol analyses will be reported and superiority will be determined only if demonstrated with the primary intention to treat analysis.

#### Methods in analysis to handle protocol non-adherence and any statistical methods to handle missing data {20c}

Careful attention will be paid to ensure that all participants are fully assessed at all time points to minimise missing data. We do not plan to use additional statistical methods such as multiple imputation as studies have demonstrated that linear mixed modelling is sufficient to control for missing data [29, 30]

#### Plans to give access to the full protocol, participant level-data and statistical code {31c}

The collated data collected by the investigators will be retained for a maximum 5 years after analysis has been completed. We will deliver a completely de-identified data set an appropriate data upon reasonable request and in agreement with the principal investigator and data protection officer.

### Oversight and monitoring

#### Composition of the coordinating centre and trial steering committee {5d}

This is a single-centre study. The trial steering committee will consist of the principal investigator (DB), trial coordinator (HK) and personnel responsible for data entry and data management (AM, CE and MBC). In addition, a research nurse employed by the institution will also be a member of this committee (HK). This committee will meet monthly to evaluate the progress of the trial, address ongoing organisational and logistical issues and consider any adverse effects.

#### Composition of the data monitoring committee, its role and reporting structure {21a}

During the process of obtaining ethical approval for this single site study, a data protection impact assessment (DPIA) screening tool was completed. This was analysed by the hospital’s data protection officer (DPO) in MMUH. It was deemed that this study posed a low risk to the rights and freedoms of natural persons and therefore a formal DPIA was not needed. Recruitment of participants is expected to be completed within less than 9 months or once the required number of participants needed is fulfilled. Due to the rapid expected inclusion of participants and the known minimal inherited risks associated with this trial, a data monitoring committee was not appointed.

#### Adverse event reporting and harms {22}

The study interventions associated with this trial does not involve any physical interventions, and all patients will receive standard of care during their perioperative period. Therefore, it is not expected that any participants that volunteer to take part in this trial will suffer from any physical complications directly related from this study. However, some participants may find the study interventions (cognitive behavioural therapy or education and mindfulness) distressing. This is not expected, but in the event if this occurs, it will be reported to the principal investigator. The participant will be removed from the trial if merited and further support will be provided.

#### Frequency and plans for auditing trial conduct {23}

A research nurse affiliated with MMUH anaesthesiology department but not involved in the trial by means patient recruitment, data collection, data entry and analysis will undertake an auditing process for trial conduct. This will occur every 3 months and would include the following: exploring the REDCap database for accuracy, proper data entry, duplicate data and adhering to data protection guidelines.

#### Plans for communicating important protocol amendments to relevant parties (e.g. trial participants, ethical committees) {25}

Approval for any study protocol amendments will be sought from the relevant IRB. Participant information leaflets will be updated accordingly and any changes to the published protocol will be reported in full in any future publications.

#### Dissemination plans {31a}

The results from this clinical trial will be fully disclosed by means of publication in an international peer-reviewed journal and by oral/poster presentations at national and international scientific meetings. Both positive and negative findings will be disclosed.

## Discussion

This paper describes the protocol of an RCT which will examine the effect of perioperative CBT on CPSP among breast cancer patients with high pain catastrophising characteristics. CPSP is a significant burden on patients that is also costly to healthcare systems [6, 31]. An intervention that has previously been shown to reduce CPSP such as the CBT [32] used in our trial may help to reduce this burden and associated costs to patients and healthcare systems globally. Our clinical trial aims to test the hypothesis that administrating a CBT intervention during the perioperative period is effective at reducing pain intensity at 3 months after breast cancer surgery, in high pain catastrophising patients. The authors will verify the aforementioned hypothesis by utilising quality controlled research methods through the use of a control group and sound statistical considerations and methods in relation to sample size, randomisation and data analysis. The use of CBT to address chronic post-surgical pain is a novel intervention in the field of surgical pain research that is highly relevant considering that up to 28% of breast cancer patients report CPSP at 3 months following surgery [3].

There are recognised limitations for our study. Whilst the CBT-CP manualised module is typically delivered over eight to ten sessions, this study has modified it to fit within four appointments, at the expense of repeated instruction and with a particular focus on challenging pain-catastrophizing cognition. We considered a number of factors when deciding on the number of CBT sessions to expose participants to. Firstly, we are conscious of minimising the research burden that we are asking of patients, at a time of heightened stress and vulnerability, without compromising the effectiveness of our treatment regimen. Secondly, it is also important to note that CBT interventions conducted over 8–10 sessions are typically designed to treat psychological disorders meeting a psychiatric diagnostic threshold as per the DSM-5 or ICD-11. They are also in manualised format to enable non-psychologists to deliver them. Our patient cohort is a non-psychiatric clinical sample, notwithstanding their heightened endorsement of pain-catastrophising, which is likely to be more modifiable over a shortened time-frame. The intervention is also being delivered by a qualified, experienced, senior psychologist. Thirdly, there is a credible amount of evidence demonstrating the efficacy of brief, non-intensive and up to three sessions of CBT interventions for clinical issues [33–35]. In addition, for the purpose of this RCT, we allowed single shot regional anaesthesia techniques (e.g. paravertebral or PECS II block) to be incorporated into the patients perioperative care, at the discretion of the treating anaesthesiologists. Some studies have suggested that implementing regional anaesthesia techniques to control acute postoperative pain may subsequently reduce the incidence of chronic post-surgical pain after breast cancer surgery [36–38]. Therefore, it can be argued that this may be an inherited limitation for our study, when examining whether CBT reduces the incidence of chronic post-surgical pain. However, more recent evidence does not support this association [39, 40]. Notably, to date, the largest RCT (*n* = 2132) examining regional anaesthesia-analgesia versus volatile general anaesthesia and opioid analgesia showed that there was no difference in frequency and severity of chronic post-surgical pain after breast cancer surgery [40]. Thus, based on these recent robust findings, we elected not to withhold regional anaesthesia techniques when examining the effectiveness of CBT on chronic post-surgical pain at 3 months after breast cancer surgery.

Our study methods endeavour to prevent bias where possible via randomisation, concealment of allocation, specified procedures and efforts to reduce incomplete data, use of a statistically appropriate sample size and consistent thorough follow-up of study patients. The study results will contribute to current evidence in surgical pain treatment and management and inform current treatment practices and standards.

## Trial status

The trial is registered on ClinicalTrials.gov Identifier: NCT04924010. The current protocol is version 2 of 05 February 2021. Participant recruitment began on 01 June 2021, and full patient recruitment is estimated to be completed by July 2022. Currently, 37.5% of patients have been recruited.

## Supplementary Information

**Additional file 1.**

## Abbreviations

BPI: Brief Pain Inventory
CBT: Cognitive behavioural therapy
CBT-CT: Cognitive Behavioural Therapy for Chronic Pain
CPSP: Chronic post-surgical pain
CRF: Case report form
DPIA: Data protection impact assessment
DPO: Data protection officer
EMT: Educational and Mindfulness Therapy
HADS: Hospital Anxiety and Depression Scale
HSE: Health Service Executive
IRB: Institutional Review Board
MMUH: Mater Misericordiae University Hospital
NRS: Numerical Pain Rating Scale
PCS: Pain Catastrophising Scale
PEM: Pain education and mindfulness
PEC: Pectoralis
QoR-15: Quality of Recovery-15
SD: Standard deviation
