# Supplementary material for: Effect of perioperative cognitive behavioural therapy on chronic post-surgical pain among breast cancer patients with high pain catastrophising characteristics: protocol for a double-blinded randomised controlled trial
Source: Trials. 2022 Jan 21;23:66. doi: 10.1186/s13063-022-06019-z (PMC8781049; doi:10.1186/s13063-022-06019-z)
Supplement: Supplementary file 1 — Additional file 1. [file 13063_2022_6019_MOESM1_ESM.pdf]

Mater Misericordiae University Hospital
Sisters of Mercy
Eccles Street, Dublin D07 R2WY, Ireland

Ospidéal Ollscoile Mater Misericordiae
Siúracha na Trócaire
Sráid Eccles, Baile Átha Cliath D07 R2WY, Éire

Tel: +353 1 8032000 Web: www.mater.ie

Not for prescription purposes

Patient Consent Form: Version 2. January 2021

Study title: Effect of perioperative cognitive behavioural therapy on chronic persistent postsurgical pain among breast cancer patients with high pain catastrophising characteristics: A randomised, double-blind clinical trial.

I have read and understood the **Patient Information Leaflet** about this research project. I have received an explanation of the nature, purpose, duration and foreseeable effects and risks of the research study, and what my involvement will be.	**Yes** ☐	**No** ☐
I have had time to consider whether to take part in this research study. My questions have been answered satisfactorily, and I have received a copy of the **Patient Information Leaflet**.	**Yes** ☐	**No** ☐
I understand that my participation is voluntary (my choice) and that I am free to withdraw at any time without my medical care or legal rights being affected.	**Yes** ☐	**No** ☐
I have to the best of my knowledge informed the investigator of my previous or present illnesses and medication, and of any consultation that I have had with a doctor for the last four months. I have not participated in any other clinical trial in the past three months.	**Yes** ☐	**No** ☐
I will contact the research investigator immediately if I suffer any unexpected or unusual symptoms because of the research study.	**Yes** ☐	**No** ☐
I am willing to allow access to my medical records by representatives of the sponsor, local or foreign regulatory authorities but understand that strict confidentiality will be maintained. The purpose of this is to check that the research study is being carried out correctly.	**Yes** ☐	**No** ☐
I agree to take part in the above research study.	**Yes** ☐	**No** ☐

-- -- ----------------

Patient Name (Block Capitals) | Signature | Date

-- -- ----------------

Name of Person taking consent | Signature | Date

(If different from doctor/researcher)

-- -- ----------------

Doctor/Researcher | Signature | Date

3 copies to be made - 1 for patient, 1 for researcher and 1 for hospital records.

Not for prescription purposes
